# Supplementary material for: COZOID: contact zone identifier for visual analysis of protein-protein interactions
Source: BMC Bioinformatics. 2018 Apr 6;19:125. doi: 10.1186/s12859-018-2113-6 (PMC5889581; doi:10.1186/s12859-018-2113-6)
Supplement: Supplementary file 2 — Software build. Executable binary file of the software tool. (ZIP 118,784 kb) [file 12859_2018_2113_MOESM2_ESM.zip › cozoid/java/docs/beansbinding-1.2.1-doc.zip!/org/jdesktop/swingbinding/JTableBinding.html]

JTableBinding (Beans Binding)


---


|  |  |  |
| --- | --- | --- |
| **Overview** \| **Package** \| **Class** \| **Use** \| **Tree** \| **Deprecated** \| **Index** \| **Help** | |  |
| **PREV CLASS**   **NEXT CLASS** | **FRAMES**    **NO FRAMES**     **All Classes** |  |
| SUMMARY: NESTED \| FIELD \| CONSTR \| METHOD | DETAIL: FIELD \| CONSTR \| METHOD |  |


---


## org.jdesktop.swingbinding Class JTableBinding<E,SS,TS>

```
java.lang.Object
  org.jdesktop.beansbinding.Binding<SS,SV,TS,TV>
      org.jdesktop.beansbinding.AutoBinding<SS,java.util.List<E>,TS,java.util.List>
          org.jdesktop.swingbinding.JTableBinding<E,SS,TS>
```

**Type Parameters:**: `E` - the type of elements in the source `List`: `SS` - the type of source object (on which the source property resolves to `List`): `TS` - the type of target object (on which the target property resolves to `JTable`)

---

``` public final class JTableBinding<E,SS,TS> extends AutoBinding<SS,java.util.List<E>,TS,java.util.List> ```

Binds a `List` of objects to act as the rows of a `JTable`.
Each object in the source `List` represents one row in the `JTable`.
Mappings from properties of the source objects to columns are created by
adding `ColumnBindings`
to a `JTableBinding`. Instances of `JTableBinding` are obtained by
calling one of the `createJTableBinding` methods in the `SwingBindings`
class.

Here is an example of creating a binding from a `List` of `Person`
objects to a `JTable`:

```
    // create the person List
    List people = createPersonList();

    // create the binding from List to JTable
    JTableBinding tb = SwingBindings.createJTableBinding(READ, people, jTable);

    // define the properties to be used for the columns
    BeanProperty firstNameP = BeanProperty.create("firstName");
    BeanProperty lastNameP = BeanProperty.create("lastName");
    BeanProperty ageP = BeanProperty.create("age");

    // configure how the properties map to columns
    tb.addColumnBinding(firstNameP).setColumnName("First Name");
    tb.addColumnBinding(lastNameP).setColumnName("Last Name");
    tb.addColumnBinding(ageP).setColumnName("Age").setColumnClass(Integer.class);

    // realize the binding
    tb.bind();
```

The `JTable` target of a `JTableBinding` acts as a live view of
the objects in the source `List`,
regardless of the update strategy (the meaning of the update strategy is
clarified later in this document). `JTableBinding`
listens to the properties specified for the `ColumnBindings`,
for all objects in the `List`, and updates the values
displayed in the `JTable` in response to change. All successful
edits made to `JTable` cell values are immediately committed back to
corresponding objects in the source `List`. If the `List` is an
instance of `ObservableList`, then changes to the `List` contents
(such as adding, removing or replacing an object) are also reflected in the
`JTable`. **Important:** Changing the contents of a non-observable
`List` while it is participating in a `JTableBinding` is unsupported,
resulting in undefined behavior and possible exceptions.

A cell in the `JTable` is editable for any given row and
column when all of the following are true: the property specified for that column
by its `ColumnBinding` is writeable for the object representing that row,
the `"editable"` property of the `JTableBinding` is `true`
(the default), and the `"editable"` property of the `ColumnBinding`
is `true` (the default).

`JTableBinding` requires
extra clarification on the operation of the
`refresh` and `save` methods and the meaning of the update
strategy. The target property of a `JTableBinding` is not the
target `JTable` property provided in the constructor, but rather a
private synthetic property representing the `List` of objects to show
in the target `JTable`. This synthetic property is readable/writeable
only when the `JTableBinding` is bound and the target `JTable`
property is readable with a `non-null` value.

It is this private synthetic property on which the `refresh` and
`save` methods operate; meaning that these methods simply cause syncing
between the value of the source `List` property and the value of the
synthetic target property (representing the `List` to be shown in the
target `JTable`). These methods do not, therefore, have anything to do
with refreshing or saving *values* in the `JTable`. Likewise, the update
strategy, which simply controls when `refresh` and `save` are
automatically called, also has nothing to do with refreshing or saving
*values* in the `JTable`.

**Note:** At the current time, the `READ_WRITE` update strategy
is not useful for `JTableBinding`. To prevent unwanted confusion,
`READ_WRITE` is translated to `READ` by `JTableBinding's`
constructor.

`JTableBinding` works by installing a custom model on the target
`JTable`, as appropriate, to represent the source `List`. The
model is installed on a target `JTable` with the first succesful call
to `refresh` with that `JTable` as the target. Subsequent calls
to `refresh` update the elements in this already-installed model.
The model is uninstalled from a target `JTable` when either the
`JTableBinding` is unbound or when the target `JTable` property
changes to no longer represent that `JTable`. Note: When the model is
uninstalled from a `JTable`, it is replaced with a `DefaultTableModel`,
in order to leave the `JTable` functional.

Some of the above is easier to understand with an example. Let's consider
a `JTableBinding` (`binding`), with update strategy
`READ`, between a property representing a `List` (`listP`)
and a property representing a `JTable` (`jTableP`). `listP`
and `jTableP` both start off readable, referring to a `non-null`
`List` and `non-null` `JTable` respectively. Let's look at
what happens for each of a sequence of events:

| Sequence | Event | Result |
| --- | --- | --- |
| 1 | explicit call to `binding.bind()` | - synthetic target property becomes readable/writeable   - `refresh()` is called   - model is installed on target `JTable`, representing list of objects |
| 2 | `listP` changes to a new `List` | - `refresh()` is called   - model is updated with new list of objects |
| 3 | `jTableP` changes to a new `JTable` | - model is uninstalled from old `JTable` |
| 4 | explicit call to `binding.refresh()` | - model is installed on target `JTable`, representing list of objects |
| 5 | `listP` changes to a new `List` | - `refresh()` is called   - model is updated with new list of objects |
| 6 | explicit call to `binding.unbind()` | - model is uninstalled from target `JTable` |

Notice that in step 3, when the value
of the `JTable` property changed, the new `JTable` did not
automatically get the model with the elements applied to it. A change to the
target value should not cause an `AutoBinding` to sync the target from
the source. Step 4 forces a sync by explicitly calling `refresh`.
Alternatively, it could be caused by any other action that results
in a `refresh` (for example, the source property changing value, or an
explicit call to `unbind` followed by `bind`).

`ColumnBindings` are managed by the `JTableBinding`. They are not
to be explicitly bound, unbound, added to a `BindingGroup`, or accessed
in a way that is not allowed for a managed binding. `BindingListeners`
added to a `ColumnBinding` are notified at the time an edited `JTable` value
is to be committed back to the source `List`. They receive notification of either
`synced` or `syncFailed`. `BindingListeners` added to the
`JTableBinding` itself are also notified of `sync` and `syncFailed`
for the `JTableBinding's ColumnBindings`.

In addition to binding the elements of a `JTable`, it is possible to
bind to the selection of a `JTable`. When binding to the selection of a `JTable`
backed by a `JTableBinding`, the selection is always in terms of elements
from the source `List`. See the list of 
interesting swing properties in the package summary for more details.

---

| **Nested Class Summary** | |
| --- | --- |
| `class` | `JTableBinding.ColumnBinding`             `ColumnBinding` represents a binding between a property of the elements in the `JTableBinding's` source `List`, and a column in the table. |

**Nested classes/interfaces inherited from class org.jdesktop.beansbinding.AutoBinding**

`AutoBinding.UpdateStrategy`

**Nested classes/interfaces inherited from class org.jdesktop.beansbinding.Binding**

`Binding.SyncFailure, Binding.SyncFailureType, Binding.ValueResult<V>`


| **Constructor Summary** | |
| --- | --- |
| `protected` | `JTableBinding(AutoBinding.UpdateStrategy strategy, SS sourceObject, Property<SS,java.util.List<E>> sourceListProperty, TS targetObject, Property<TS,? extends javax.swing.JTable> targetJTableProperty, java.lang.String name)`             Constructs an instance of `JTableBinding`. |


| **Method Summary** | |
| --- | --- |
| `JTableBinding.ColumnBinding` | `addColumnBinding(int index, Property<E,?> columnProperty)`             Creates a `ColumnBinding` and inserts it at the given index into the list of `ColumnBindings` maintained by this `JTableBinding`. |
| `JTableBinding.ColumnBinding` | `addColumnBinding(int index, Property<E,?> columnProperty, java.lang.String name)`             Creates a `ColumnBinding` and inserts it at the given index into the list of `ColumnBindings` maintained by this `JTableBinding`. |
| `JTableBinding.ColumnBinding` | `addColumnBinding(Property<E,?> columnProperty)`             Creates a `ColumnBinding` and adds it to the end of the list of `ColumnBindings` maintained by this `JTableBinding`. |
| `JTableBinding.ColumnBinding` | `addColumnBinding(Property<E,?> columnProperty, java.lang.String name)`             Creates a named `ColumnBinding` and adds it to the end of the list of `ColumnBindings` maintained by this `JTableBinding`. |
| `protected  void` | `bindImpl()`             Called by `Binding.bind()` to allow subclasses to initiate binding. |
| `JTableBinding.ColumnBinding` | `getColumnBinding(int index)`             Returns the `ColumnBinding` with the given index in the list maintained by this `JTableBinding`. |
| `java.util.List<JTableBinding.ColumnBinding>` | `getColumnBindings()`             Returns an unmodifiable copy of the list of `ColumnBindings` maintained by this `JTableBinding`. |
| `boolean` | `isEditable()`             Returns whether or not the cells of the table should be editable. |
| `JTableBinding.ColumnBinding` | `removeColumnBinding(int index)`             Removes the `ColumnBinding` with the given index from the list maintained by this `JTableBinding`. |
| `boolean` | `removeColumnBinding(JTableBinding.ColumnBinding binding)`             Removes the given `ColumnBinding` from the list maintained by this `JTableBinding`. |
| `void` | `setEditable(boolean editable)`             Sets whether or not the cells of the table should be editable. |
| `protected  void` | `unbindImpl()`             Called by `Binding.unbind()` to allow subclasses to uninitiate binding. |

**Methods inherited from class org.jdesktop.beansbinding.AutoBinding**

`getUpdateStrategy, paramString, sourceChangedImpl, targetChangedImpl`

**Methods inherited from class org.jdesktop.beansbinding.Binding**

`addBindingListener, addPropertyChangeListener, addPropertyChangeListener, bind, bindUnmanaged, firePropertyChange, getBindingListeners, getConverter, getName, getPropertyChangeListeners, getPropertyChangeListeners, getSourceNullValue, getSourceObject, getSourceProperty, getSourceUnreadableValue, getSourceValueForTarget, getTargetNullValue, getTargetObject, getTargetProperty, getTargetValueForSource, getValidator, isBound, isManaged, isSourceUnreadableValueSet, notifySynced, notifySyncFailed, refresh, refreshAndNotify, refreshAndNotifyUnmanaged, refreshUnmanaged, removeBindingListener, removePropertyChangeListener, removePropertyChangeListener, save, saveAndNotify, saveAndNotifyUnmanaged, saveUnmanaged, setConverter, setManaged, setSourceNullValue, setSourceObject, setSourceObjectUnmanaged, setSourceProperty, setSourceUnreadableValue, setTargetNullValue, setTargetObject, setTargetObjectUnmanaged, setTargetProperty, setValidator, throwIfBound, throwIfManaged, throwIfUnbound, toString, unbind, unbindUnmanaged, unsetSourceUnreadableValue`

**Methods inherited from class java.lang.Object**

`clone, equals, finalize, getClass, hashCode, notify, notifyAll, wait, wait, wait`

**Constructor Detail**

### JTableBinding

```
protected JTableBinding(AutoBinding.UpdateStrategy strategy,
                        SS sourceObject,
                        Property<SS,java.util.List<E>> sourceListProperty,
                        TS targetObject,
                        Property<TS,? extends javax.swing.JTable> targetJTableProperty,
                        java.lang.String name)
```

:   Constructs an instance of `JTableBinding`.

    **Parameters:**: `strategy` - the update strategy: `sourceObject` - the source object: `sourceListProperty` - a property on the source object that resolves to the `List` of elements: `targetObject` - the target object: `targetJTableProperty` - a property on the target object that resolves to a `JTable`: `name` - a name for the `JTableBinding` **Throws:**: `java.lang.IllegalArgumentException` - if the source property or target property is `null`


**Method Detail**

### bindImpl

```
protected void bindImpl()
```

:   **Description copied from class: `Binding`**
:   Called by `Binding.bind()` to allow subclasses to initiate binding.
    Subclasses typically need not install `PropertyStateListeners`
    on the source property and target property as they will be notified
    by calls to `Binding.sourceChangedImpl(org.jdesktop.beansbinding.PropertyStateEvent)` and `Binding.targetChangedImpl(org.jdesktop.beansbinding.PropertyStateEvent)`
    when the source and target properties change respectively.

    :   **Overrides:**: `bindImpl` in class `AutoBinding<SS,java.util.List<E>,TS,java.util.List>`
    :   **See Also:**: `Binding.unbindImpl()`

---


### unbindImpl

```
protected void unbindImpl()
```

:   **Description copied from class: `Binding`**
:   Called by `Binding.unbind()` to allow subclasses to uninitiate binding.

    :   **Overrides:**: `unbindImpl` in class `AutoBinding<SS,java.util.List<E>,TS,java.util.List>`
    :   **See Also:**: `Binding.bindImpl()`

---


### setEditable

```
public void setEditable(boolean editable)
```

:   Sets whether or not the cells of the table should be editable.
    The default for this property is `true`.
    See this paragraph in the class level
    documentation on editability.

    :   **Parameters:**: `editable` - whether or not the cells of the table should be editable

---


### isEditable

```
public boolean isEditable()
```

:   Returns whether or not the cells of the table should be editable.
    The default for this property is `true`.
    See this paragraph in the class level
    documentation on editability.

    :   **Returns:**: whether or not the cells of the table should be editable

---


### addColumnBinding

```
public JTableBinding.ColumnBinding addColumnBinding(Property<E,?> columnProperty)
```

:   Creates a `ColumnBinding` and adds it to the end of the list of `ColumnBindings`
    maintained by this `JTableBinding`.

    The list of `ColumnBindings` dictates the columns to be displayed in the
    `JTable`, with a `ColumnBinding's` order in the list determining its
    table model index.

    :   **Parameters:**: `columnProperty` - the property with which to derive cell values from the elements of the source `List` **Returns:**: the `ColumnBinding` **Throws:**: `java.lang.IllegalArgumentException` - if `columnProperty` is `null` **See Also:**: `JTableBinding.ColumnBinding`

---


### addColumnBinding

```
public JTableBinding.ColumnBinding addColumnBinding(Property<E,?> columnProperty,
                                                    java.lang.String name)
```

:   Creates a named `ColumnBinding` and adds it to the end of the list of `ColumnBindings`
    maintained by this `JTableBinding`.

    The list of `ColumnBindings` dictates the columns to be displayed in the
    `JTable`, with a `ColumnBinding's` order in the list determining its
    table model index.

    :   **Parameters:**: `columnProperty` - the property with which to derive cell values from the elements of the source `List`: `name` - a name for the column binding **Returns:**: the `ColumnBinding` **Throws:**: `java.lang.IllegalArgumentException` - if `columnProperty` is `null` **See Also:**: `JTableBinding.ColumnBinding`

---


### addColumnBinding

```
public JTableBinding.ColumnBinding addColumnBinding(int index,
                                                    Property<E,?> columnProperty)
```

:   Creates a `ColumnBinding` and inserts it at the given index into the list
    of `ColumnBindings` maintained by this `JTableBinding`.

    The list of `ColumnBindings` dictates the columns to be displayed in the
    `JTable`, with a `ColumnBinding's` order in the list determining its
    table model index.

    :   **Parameters:**: `index` - the index at which to insert the `ColumnBinding`: `columnProperty` - the property with which to derive cell values from the elements of the source `List` **Returns:**: the `ColumnBinding` **Throws:**: `java.lang.IllegalArgumentException` - if `columnProperty` is `null` **See Also:**: `JTableBinding.ColumnBinding`

---


### addColumnBinding

```
public JTableBinding.ColumnBinding addColumnBinding(int index,
                                                    Property<E,?> columnProperty,
                                                    java.lang.String name)
```

:   Creates a `ColumnBinding` and inserts it at the given index into the list
    of `ColumnBindings` maintained by this `JTableBinding`.

    The list of `ColumnBindings` dictates the columns to be displayed in the
    `JTable`, with a `ColumnBinding's` order in the list determining its
    table model index.

    :   **Parameters:**: `index` - the index at which to insert the `ColumnBinding`: `columnProperty` - the property with which to derive cell values from the elements of the source `List`: `name` - a name for the `ColumnBinding` **Returns:**: the `ColumnBinding` **Throws:**: `java.lang.IllegalArgumentException` - if `columnProperty` is `null` **See Also:**: `JTableBinding.ColumnBinding`

---


### removeColumnBinding

```
public boolean removeColumnBinding(JTableBinding.ColumnBinding binding)
```

:   Removes the given `ColumnBinding` from the list maintained
    by this `JTableBinding`.

    The list of `ColumnBindings` dictates the columns to be displayed in the
    `JTable`, with a `ColumnBinding's` order in the list determining its
    table model index.

    :   **Parameters:**: `binding` - the `ColumnBinding` to remove **See Also:**: `addColumnBinding(Property, String)`

---


### removeColumnBinding

```
public JTableBinding.ColumnBinding removeColumnBinding(int index)
```

:   Removes the `ColumnBinding` with the given index from the list maintained
    by this `JTableBinding`.

    The list of `ColumnBindings` dictates the columns to be displayed in the
    `JTable`, with a `ColumnBinding's` order in the list determining its
    table model index.

    :   **Parameters:**: `index` - the index of the `ColumnBinding` to remove **See Also:**: `addColumnBinding(Property, String)`

---


### getColumnBinding

```
public JTableBinding.ColumnBinding getColumnBinding(int index)
```

:   Returns the `ColumnBinding` with the given index in the list maintained
    by this `JTableBinding`.

    The list of `ColumnBindings` dictates the columns to be displayed in the
    `JTable`, with a `ColumnBinding's` order in the list determining its
    table model index.

    :   **Parameters:**: `index` - the index of the `ColumnBinding` to return **Returns:**: the `ColumnBinding` at the given index **See Also:**: `addColumnBinding(Property, String)`

---


### getColumnBindings

```
public java.util.List<JTableBinding.ColumnBinding> getColumnBindings()
```

:   Returns an unmodifiable copy of the list of `ColumnBindings` maintained
    by this `JTableBinding`.

    The list of `ColumnBindings` dictates the columns to be displayed in the
    `JTable`, with a `ColumnBinding's` order in the list determining its
    table model index.

    :   **Returns:**: the list of `ColumnBindings` **See Also:**: `addColumnBinding(Property, String)`


---


|  |  |  |
| --- | --- | --- |
| **Overview** \| **Package** \| **Class** \| **Use** \| **Tree** \| **Deprecated** \| **Index** \| **Help** | |  |
| **PREV CLASS**   **NEXT CLASS** | **FRAMES**    **NO FRAMES**     **All Classes** |  |
| SUMMARY: NESTED \| FIELD \| CONSTR \| METHOD | DETAIL: FIELD \| CONSTR \| METHOD |  |


---
